# Operation Remote Immunity: exploring the impact of a service-learning elective in remote Indigenous communities

**DOI:** 10.1186/s12909-023-04434-7

**Published:** 2023-06-20

**Authors:** Hannah Mikhail, Brenton Button, Joseph LeBlanc, Catherine Cervin, Erin Cameron

**Affiliations:** 1 Northern Ontario School of Medicine University in Sudbury, 935 Ramsey Lake Rd, Sudbury, ON P3E 2C6 Canada; 2 Northern Ontario School of Medicine University in Thunder Bay, 955 Oliver Rd, Thunder Bay, P7B 5E1 Canada; 3 University of Winnipeg, 515 Portage Avenue, Winnipeg, MB R3B 2E9 Canada

**Keywords:** Medical education, Service-learning, Medical learners, Indigenous health, COVID vaccine

## Abstract

**Background:**

The novel coronavirus, COVID-19, emerged in December 2019. Shortly after, vaccines against the virus were distributed in Canada for public use, but the remoteness of many northern Indigenous communities in Ontario posed a challenge for vaccine distribution and dissemination. The Ministry of Health partnered with the Northern Ontario School of Medicine University (NOSMU) and the air ambulance service, Ornge, to assist in delivering the vaccination doses to 31 fly-in communities in the Nishnawbe Aski Nation and Moosonee, all within Ontario. These deployments were considered “service-learning electives” for Undergraduate and Postgraduate medical learners from NOSMU who joined the operation in two-week deployments. NOSMU is renowned for its social accountability mandate and gives its medical learners opportunities to participate in service-learning to enhance their medical skills and cultural sensitivity. The purpose of this study is to examine the relationship between social accountability and medical learners’ experiences during a service-learning elective in northern Indigenous communities in Ontario during the COVID-19 pandemic.

**Methods:**

Data were collected through a planned post-placement activity completed by eighteen Undergraduate and Postgraduate medical learners, who participated in the vaccine deployment. The activity consisted of a 500-word reflective response passage. Thematic analysis was used to identify, analyze, and report the themes within the collected data.

**Results:**

Two themes were identified by the authors, which formed a concise overview of the collected data: (1) confronting the realities of working in Indigenous communities; and (2) service-learning as a path to social accountability.

**Conclusions:**

These vaccine deployments were an opportunity for medical learners to engage in service-learning and engage with Indigenous communities in Northern Ontario. Service-learning is an exceptional method which provides an opportunity to expand knowledge on the social determinants of health, social justice, and social accountability. The medical learners in this study reiterated the idea that learning medicine through a service-learning model leads to a greater depth of knowledge on Indigenous health and culture, and enhances medical knowledge compared to classroom learning.

## Background

The first case of the novel coronavirus, also known as COVID-19 or SARS-CoV-2, was identified in humans in December 2019, with the first case in Northern Ontario occurring in April 2020 [1]. During the following year, the virus caused great devastation in every country causing global shutdowns, economic instability, and significant mortality [2]. The first case of COVID-19 in Northern Ontario occurred in April 2020 [3] and further revealed the extent of health disparities worldwide as certain groups of people were disproportionately affected by the pandemic [3]. The pandemic has had ongoing negative effects on mental health, resource allocations and healthcare services. In December 2020, the first COVID-19 vaccine was authorized for use in Canada [4]; however, the remoteness of many northern Indigenous communities in Ontario posed a challenge for the rollout of the two-step vaccine [5]. Remote communities have limited healthcare professionals and facilities to assist with vaccine distribution [5], and the traumatic history between Indigenous Peoples and the westernized healthcare system instilled much mistrust in some Indigenous communities [6].

Numerous factors have led to this complex relationship between Indigenous Peoples, the federal government and westernized healthcare [7]. Colonization, for example, has had traumatic intergenerational effects on the overall well-being of Indigenous populations in Canada [7]. Many studies have linked residential schooling to poorer general health, including increased rates of chronic and infectious diseases, substance abuse, and mental health disorders [7]. Colonization has led to a loss of culture, language, traditional ceremonies, and family structures for many Indigenous communities [8, 9]. To provide culturally safe care during the pandemic and to ensure the safety of these northern communities, the Ontario government partnered with the air ambulance service Ornge as the lead, and Nishnawbe Aski Nation as the co-lead to develop Operation Remote Immunity (ORI). The purpose of ORI was to offer full COVID-19 immunization to 31 remote fly-in communities in the Nishnawbe Aski Nation and Moosonee by mid-April 2021 [10]. The Northern Ontario School of Medicine University (NOSMU) was one of the medical schools recruited to ORI as a partner to provide medical professionals and learners to join the vaccination teams [10].

NOSMU is a medical university in Ontario, with campuses in Sudbury and Thunder Bay, that has a social accountability mandate to educate medical learners with a goal for them to contribute to healthcare in northern Ontario’s urban, rural and remote communities [11]. The mandate expresses an obligation for NOSMU to focus their education, research, and service activities towards the priority health concerns of the communities and population groups of Northern Ontario [12]. Current priority health concerns in Northern Ontario include providing healthcare for underserviced regions, reducing health inequities, advocating for and providing high quality care for Indigenous and Francophone populations, and reducing the shortage of physicians and other allied health professionals in the region [12]. NOSMU is working to achieve this social accountability mandate by accepting medical learners who have a rural or northern background and upbringing, and creating on-going relationships with Indigenous communities in Northern Ontario [12]. NOSMU encourages faculty proficiency in Indigenous health, and promotes the hiring of Indigenous faculty members [13]. The medical university has utilized a community engagement model, where Indigenous partner communities actively participate in shaping and delivering the medical curriculum by means of a one-month immersive placement in an Indigenous community for first year Undergraduate NOSMU students [13, 14]. There are also opportunities for third year medical learners to gain experiences at Indigenous Health Authorities and Health Centres. These learning experiences emphasize Indigenous health and cultural safety, which medical learners can carry into their future practices [14]. These relationships with NOSMU and Indigenous communities potentially fostered more trusting relationships during Operation Remote Immunity and allowed the service-learning elective to occur.

An effective method of teaching the importance of social accountability is through service-learning. Service-learning is a structured experience where medical learners take part in clinical and community experiences first-hand to develop their knowledge, skills, and values [15]. An important aspect of service-learning is continuous reflection throughout the experience. During these experiences, learners tend to be actively engaged, developing their problem-solving and critical thinking skills, which promotes personal reflection and growth [15]. Service-learning goes hand-in-hand with social accountability, as it is an effective way to directly contribute to one’s community and service priority population groups [15]. Service-learning allows medical learners to enhance their knowledge on specific population groups and develop their skills to a greater degree in order to provide culturally and medically safe care [15]. Previous research has shown that service-learning promotes higher levels of retention in the communities the learners work in. It also promotes altruism and social humility in learners and encourages continued work in underserviced regions [16].

The purpose of this study is to examine the relationship between social accountability and medical learners’ experiences during a service-learning elective in remote First Nations, accessible only by air, water, or ice road, during the COVID-19 pandemic. This is a critical area of learning and research, as providing high-quality learning opportunities for Canada’s future physicians in an area of such importance will hopefully lead to greater levels of social accountability and culturally appropriate care for Indigenous populations in Canada. In addition, efforts must be made to reduce the health inequities that exist between Indigenous and non-Indigenous Canadians, and that reconciliation with Indigenous Peoples of Canada is prioritized.

## Methods

### Service-Learning Elective

During the COVID-19 vaccine rollout in early 2020, medical learners at NOSMU had an opportunity to take part in the ORI service-learning elective. This elective involved assisting in the administration of the Moderna COVID-19 vaccine across northern remote First Nations in the Nishnawbe Aski Nation and Moosonee. Ornge, Ontario’s air ambulance service, was the provincial lead to distribute the COVID-19 Moderna vaccine to a portion of remote First Nations in Northern Ontario. NOSMU was recruited by Ornge to assist with the vaccine deployment and administration by supplying teams of medical personnel, which included NOSMU Faculty as well as Undergraduate and Postgraduate medical learners, who rotated through 2-week blocks. These medical learners were required to complete cultural sensitivity training prior to deployment, which consisted of pre-readings, a minimum of 11 h of the San’yas cultural safety and sensitivity modules, and develop learning outcomes based on the CanMEDs competencies. The CanMEDs competencies is a framework outlining the necessary skills that a physician requires to effectively meet the health needs of their patients. The competencies include Medical Expert, Communicator, Collaborator, Leader, Health Advocate, Scholar and Professional. For the learners, the ORI experience was considered a service-learning elective focusing on delivering real-time culturally sensitive care to First Nations populations in northern Canada.

### Data collection

Data were collected through a planned post-service-learning activity. The activity was designed by a team of content experts, instructional designers and NOSMU faculty members. The activity consisted of a 500-word reflective response passage written by the medical learners who participated in ORI, focusing on key learning experiences and take-aways from the service-learning elective. Their 500-word responses were guided by questions that focused on topics relevant to the overarching research topic such as social accountability, advocacy, and how the experience reinforced their prior learnings. The responses were anonymized by administrators at NOSMU, and the data were analyzed for themes. Ethics for this project was waived by Lakehead University Research Ethics Board as data met the criteria of the Tri-Council Policy Statement 2, Chapter 2, Article 2.4.

### Participants

Participants consisted of eighteen Undergraduate and Postgraduate medical learners from the Northern Ontario School of Medicine who took part in this service-learning elective.

### Data analysis

Thematic analysis was used to identify, analyze, and report the themes within the collected data. Initially, the 500-word written reflections were actively read by the lead author and examined for meanings and patterns [17]. Once an in-depth understanding of each passage was gained, the lead author began generating preliminary codes from the data, from two rounds of coding the raw data. These codes were organized into a codebook to improve the rigor of the data analysis. The codes outlined the most basic foundations of the data that were relevant to the primary research focus [17]. The codes themselves were then analyzed and arranged into encompassing themes. These themes were reviewed at the level of the coded data, to ensure that they formed an articulate pattern that related to the primary research focus. Finally, the themes were refined and confirmed by a second author on this paper and were named. The final themes provide a succinct, coherent explanation of the data.

The lead author HM is a Caucasian female medical student at NOSMU and she is in her mid 20 s. She coded, analyzed and interpreted the results based on her experiences learning about Indigenous health and health inequities in a Master of Public Health program at Queen’s University, where she published one qualitative study, and during her studies and clinical experiences at NOSMU. The second author BB is a Caucasian man who helped design the project, participated in coding and analyzing of the reflections, and helped critically edit the paper. He is an Assistant Professor in a faculty of education and part of his research focuses on pedagogy. He has experience in both qualitative and quantitative data analysis. The third author JL contributed to the analysis and interpretation of the data providing an Indigenous viewpoint, and helped critically edit the manuscript based on his interpretations as an Indigenous man. He is currently the Associate Dean of Equity and Inclusion at NOSMU and was the previous Director of Indigenous Affairs at NOSMU since 2018. The fourth author CC is a Caucasian woman who helped to coordinate between the NOSMU elective programs and the research team. She aided in interpreting the data and in critically editing the manuscript. The final author EC is a Caucasian woman who helped design the study and critically edit the manuscript. She is the director of the Center for Social Accountability, an Associate Professor at NOSMU, and an educational scholar. She is the founder and lead investigator of the Medical Education Research Lab in the North (MERLIN). It should be addressed that the lead author and critical editor of the manuscript are not Indigenous but had numerous discussions with and guidance on the analyses of the data from JL.

## Results

In the data, two main themes were identified across all data: (1) confronting the realities of working in Indigenous communities; and (2) service-learning as a path to social accountability. There were no differences in the results of the data across the Undergraduate and Postgraduate medical levels.

### Theme 1: Confronting the realities of working in Indigenous communities

The written reflections focused on the health and well-being of the Indigenous populations living in the remote communities. Overall, the participants focused on four subthemes: the social determinants of health, the on-going effects of colonialism, mistrust in western institutions of education and medicine, and vaccine hesitancy. The medical learners reflected on how experiencing the inequities in the social determinants of health first-hand allowed them to understand the negative impact that these inequities have on health to a much greater degree than learning in classrooms. One learner described: “The reality of extremely high food prices was made clear when community members commented that the case of twenty-four water bottles that our team brought each day would cost approximately fifty dollars at the local store” (Response 18). Learners discussed the ongoing effects of colonialism in Canada and subsequently how they would be perceived within the community when providing westernized healthcare: “… I was worried about how this initiative would be perceived by the communities, given our country’s history…” (Response 2). They discussed how ongoing colonialism has manifested in justified mistrust in westernized healthcare and vaccine hesitancy for Indigenous Peoples in Ontario: “… Someone was concerned with the possibility to be injected with a microchip… I wonder if this belief stemmed from the mistrust of past trauma and colonization.” (Response 3). The subthemes are further described in Table 1. See Table 1.

**Table 1:** Four subthemes within Theme 1 – Confronting the realities of working in Indigenous communities

Description of subtheme:	Representative participant quote:
**Social determinants of health**	
Many of the written reflections touched on how this service-learning elective provided first-hand observations of the inequities in the social determinants of health for Indigenous communities and how that is impacting the overall health of this population:	*“Continuously I have heard of the struggles that the Indigenous communities face, however, it is not until you visualize and experience the obstacles first-hand that you really start to realize how impactful they are” (Response 4)*
The social determinants of health that many of the participants mentioned witnessing first-hand in the remote communities included inadequate housing, restricted access to clean drinking water and healthy food, winter road access, inaccessible health services and health professionals, geographical isolation and limited educational opportunities:	*“Home visits were unique experiences, as I got to see first-hand the housing challenges that I had only previously read about. Crowded and multi-generational houses were common due to housing shortages, with many people living outside in tents either by choice or necessity” (Response 8)*
**On-going effects of colonialism**	
Participants spoke of Canada’s traumatic experience of colonialism between Indigenous Peoples and settlers. They acknowledged that distressing impacts of colonization and racism exist:	*“I have come to realize first-hand the real and actual impact of colonization on this population as well as its direct consequences on healthcare” (Response 3)*
One participant was concerned about how community members would perceive the teams entering the communities to dispense and distribute the COVID-19 vaccines:	*“Prior to my deployment, I was worried about how this initiative would be perceived by the communities, given our country’s history…” (Response 2)*
Many of the reflections mirrored the sentiment that there are residual and continuous effects of colonialism in many of the remote communities, such as a general mistrust in western medicine. Some of the participants reflected on the fact that Indigenous Peoples were subjected to experimentation and non-consensual medical procedures in Canadian hospitals, residential and day schools, and in child protective services:	*“There are many well-known examples such as tuberculosis sanatoriums, forced sterilization and nutrition experiments in residential schools. Of course these patients would be hesitant about this vaccine roll out and when reflecting on this history, the fear and skepticism only makes sense” (Response 12)*
**Mistrust in western institutions of education and medicine**	
When the mistrust in western medicine was present, it manifested in some of the community members’ responses to the COVID-19 vaccine:	*“… someone was concerned with the possibility to be injected with a microchip. They believed a black part of the syringe was injected into their arm…” (Response 3)*
**Vaccine hesitancy**	
Finally, vaccine hesitancy arose in the reflections as a concern in some of the fly-in communities during ORI. One of the most prevalent causes for this vaccine hesitancy was a mistrust in western medicine as stated, but another common explanation was differing acceptance of leadership in each community:	*“Vaccine hesitancy was a bigger challenge than I anticipated… The chief usually attracts respect from other community members, and I noticed an increased vaccine uptake when the chief was personally vaccinated, [and] promoted the vaccine *via* mainstream channels and the radio” (Response 3)*
The written reflections stated that vaccine hesitancy also seemed to vary depending on each community’s lived experience with COVID-19:	*“One community had some relative hesitancy… Other communities had been directly affected by COVID recently, with members from the community testing positive… both communities had very different lived experiences with the pandemic, and different views on what the vaccine meant for their community” (Response 15)*
Finally, a few reflections focused on how some vaccine hesitancy within the communities stemmed from false information read from social media websites:	*“…false information is just as easy to come by as evidence-based information is. For example, many communities had preconceived notions around the COVID-19 vaccine due to scam information posted on multiple social media platforms” (Response 4)*

### Theme 2: Service-learning as a path to social accountability

The value of this service-learning experience was well represented in the written reflections, and centered on four subthemes: clinical courage, learning about social accountability and cultural sensitivity, promoting future practice in the north, and understanding the clinical importance of the CanMEDs competencies. This theme demonstrated the utility of service-learning in providing high-quality educational experiences for medical learners, which they can carry into their professional careers. Learners had the ability to gain confidence in their clinical skills and practice outside of their usual scope at an earlier stage in their learning: “As a first-year medical student, the very first vaccine I ever administered was the COVID-19 vaccine… that is something I will always remember” (Response 8). The medical learners had a unique opportunity to reflect on how learning in-person advanced their knowledge on cultural safety, humility and sensitivity, and how that could promote social accountability when caring for their patients in the future. One learner reflected: “… I worked on providing culturally safe care and engaged in extensive self-reflection on how my own non-Indigenous culture could promote unconscious bias or stereotypes which could hinder my ability to provide equitable care to these community members” (Response 10). Numerous medical learners discussed how having these experiences early on in their medical education will shape the direction their future careers will take and that they will carry these values on: “In my future practice I want to bring this baggage of knowledge I was fortunate to develop to allow me to provide culturally safe and accessible care to Indigenous populations…” (Response 3). The responses focused on how the CanMEDs competencies of Communication and Collaboration were integral for the team members to exhibit in order for this service-learning elective to succeed in providing socially accountable healthcare: “The most striking aspect of this experience was the team-based collaborative approach to vaccine administration that allowed for empowerment of these Indigenous communities” (Response 5). The subthemes are further described in Table 2. See Table 2.

**Table 2:** Four subthemes within Theme 2 – Service-learning as a path to social accountability

Description of subtheme:	Representative participant quote:
**Clinical courage**	
The participants conveyed how much they learned in terms of clinical skills and medical knowledge during this service-learning experience. Many of them mentioned that they gained confidence and skills and courage that they never would have learned during classroom learning:	*“When I was offered the chance to give a vaccine, I quickly took it. I was so scared before deployment about having to give one, because I had never done it before” (Response 7)*
Many participants stated the fact that learning during an in-person experiences accelerates learning and develops skills:	*“The added responsibility of acting as an educator for a patient… has given me more confidence going forward in communicating with patients” (Response 1)*
**Learning social accountability and cultural sensitivity**	
Participants recalled many experiences where they were taught the importance of culturally competent care, cultural safety, and social accountability in medicine, specifically for Indigenous populations:	*“… I also worked on providing culturally safe care and engaged in extensive self-reflection, both on how the history of colonialism and oppression of Indigenous peoples presented itself with vaccine hesitancy, as well as on how my own non-Indigenous culture could promote unconscious bias or stereotypes which could hinder my ability to provide equitable care to these community members” (Response 8)*
Many of the written reflections focused on the importance of providing culturally safe care, and not assuming or forcing western views onto Indigenous patients:	*“What was invaluable was learning that history is absolutely an important factor in healthcare and its delivery. We have to not only be culturally sensitive, but historically informed to provide well-rounded and sensitive care to Indigenous communities. We should always address hesitancies with an open mind and be informed of history as it relates to Indigenous people and western medicine” (Response 12)*
**Promoting future practice in the north**	
Included in NOSMU’s mandate is to address the health human resource needs of Northern Ontario and have its medical learners remain in the north and serve the needs of northern communities. Many participants reflected that this service-learning experience encouraged them to return to the remote communities from ORI, and do locum work as a part of their future medical practice, or work full-time in these communities:	*“This entire experience has reminded me so much about what I hope to accomplish in my lifetime. It has reminded me of where I want to work, and why. It has re-inspired my desire to work and locum in [Information redacted]… It’s a privilege to have the opportunity to do this work someday, I am grateful for that” (Response 15)*
Participants stated that they wanted to take what they had learned from this experience and apply it to their future medical practices, in order to foster a culturally safe and appropriate practice for their Indigenous patients:	*“This experience… has also added much needed context to my view of Indigenous culture and health, which will guide me in providing more holistic and appropriate care to this population… I hope to use my platform as a future physician to work as an ally, supporting Indigenous leaders in advocating for improved access to equitable, self-determined healthcare” (Response 5)*
**Understanding the clinical importance of the CanMEDs competencies**	
This service-learning elective also provided a unique opportunity to apply the CanMEDs competencies and understand the clinical utility of them in medicine and social accountability, rather than just learning them in a classroom. Two CanMEDs competencies that stood out in the reflections were collaboration and communication. Collaboration within the service-learning elective was a competency that was commonly reflected on, as the deployment groups consisted of a wide range of allied health professionals:	*“I feel fortunate to have had the opportunity to work as part of a multi-disciplinary deployment team. Led by paramedics, we worked alongside other medical students, residents, physicians and nurses… By collaborating with a multidisciplinary team, I feel I have better developed my transferable collaboration skills” (Response 1)*
Participants made it evident that the success of the operation also hinged on respectful collaboration with Indigenous community members during the service-learning elective:	*“I feel that ORI is an excellent example of health advocacy and interdisciplinary collaboration. One key aspect of this historical initiative was the collaboration and partnerships that were developed with the community liaisons. They played a vital role in welcoming us into the community, booking appointments, and ensuring that there were always the perfect number of people coming to the clinic to ensure that there were no vaccine doses wasted” (Response 2)*
Another participant reiterated the importance of communication with patients, as well as whole communities, and including them in the entire decision-making process:	*“My takeaway was that we cannot always predict why or how patients/communities will react to our interventions and that it is important to include them in decision-making” (Response 10)*
A final meaningful element of the reflections was the value of communication and its significance in social accountability and medicine. Participants spoke about the importance of all aspects of communication, such as non-verbal communication:	*“A technique I have utilized to dissipate myths associated with the COVID-19 vaccine was to listen actively. By doing so I was able to understand their concerns and where the individual’s beliefs were coming from” (Response 3)*

## Discussion

The purpose of this paper was to examine the relationship between social accountability and the experiences of medical learners during a service-learning elective in northern Indigenous communities in Ontario, during the COVID-19 pandemic. Across Undergraduate and Postgraduate medical learner levels, the reflections largely focused on two main themes. The first theme encompassed how the medical learners confronted the realities of working in Indigenous communities, by reflecting on the social determinants of health, the impacts of colonialism on Indigenous health, mistrust in western medicine, and vaccine hesitancy. The second theme was how service-learning is a path to encouraging social accountability, in which the learners reflected on how service-learning promotes clinical courage, clarifies the importance of social accountability and cultural sensitivity, encourages northern work in future medical practice, and teaches about the importance of the CanMEDs competencies in clinical practice. The results from this study support the importance and utility of service-learning for medical learners. Service-learning is an effective educational tool for becoming knowledgeable on the importance of social accountability in the community, which the learners can then carry into their future practices as physicians.

From the data it is apparent that this service-learning elective influenced participants’ understanding of Indigenous health. Indigenous Peoples in Canada have, and still are, experiencing social, economic, and health disparities as a result of colonialism [18]. These inequities are evident in terms of the social determinants of health, as Indigenous communities currently face higher rates of food insecurity, inadequate housing, and insufficient access to healthcare compared to non-Indigenous Canadians [18]. Immersive, in-person service-learning provides medical learners with an opportunity to increase their awareness of inequities and develop their knowledge and advocacy skills, compared to classroom learning [19]. Service-learning has been shown to allow medical learners to remain altruistic throughout their learning and careers [16]. This method of education provides in-person experiences that can improve learners’ relationships with individual communities, allows them to consider community context in relation to care models provided, allows learners to experience the social determinants of health first-hand, and they can witness the blurring of the medical hierarchy [16]. All of which may increase social accountability in these rural northern communities and allow better healthcare, compared to classroom learning models. During this elective the medical learners spent very little time in the community, spending most of their time in the vaccination centres and rarely staying overnight in the remote communities. Although the short-term service-learning elective did seem to promote an understanding of health inequities in these remote Indigenous communities, learners must be afforded a more immersive placement opportunity to develop a more in-depth understanding of the inequities faced by Indigenous peoples.

The traumatic effects of colonialism on Indigenous populations were acknowledged in the written reflections. The medical learners spoke about the impact that colonialism has had on Indigenous health and well-being and were apprehensive about how they would be perceived entering the communities to dispense the COVID-19 vaccines. When referring to colonialism, many of the medical learners spoke about it as a historic event, but colonialism is still ever-present in Canadian society. One example is the federal government’s goal to end all drinking water advisories in First Nations communities by mid-2021, which has not been achieved to date [20]. Having access to clean and reliable drinking water is a basic human right that is still denied to many First Nation communities in Canada [21]. The federal government has been complacent in failing to rectify this environmental justice issue [21]. The government’s ability to disregard this egregious human rights issue is an example of current-day colonialism. Although the medical learners acknowledged the devastating effects that colonialism has had on Indigenous communities in the past, it is necessary to recognize that colonialism and systemic racism continue today. It is essential that Canadian medical schools refer to colonialism as a present-day issue as it will lead to more culturally safe healthcare for Indigenous patients.

The medical learners often reflected on how some community members were experiencing a mistrust in western medicine. Canada has a long history of conducting unethical experiments on Indigenous adults and children, as elders can recount being subjected to medical experimentation against their will when they were children [22]. These experimentations include nutrition experiments, forced sterilization, and tuberculosis vaccine testing [22]. This unwilling subjection to previous medical experimentation likely has a role in the hesitancy to receive the COVID-19 vaccine [22]. The concerns and fears that Indigenous people have regarding the vaccine need to be taken into serious consideration and separated from the anti-vaccination movement. One method of increasing vaccine uptake in communities that are vaccine hesitant is to engage influential community leaders in public service announcements and educational initiatives about the vaccine [23]. Leadership creates an environment where community members can feel safer and more confident in their decisions. A long-time leader in Indigenous health and current New Democratic Party Member of Ontario Parliament, Sol Mamakwa, took part in a vaccine campaign in Muskrat Dam First Nation and received his COVID-19 vaccine publicly in hopes of combatting the vaccine hesitancy in First Nation communities [24]. Two weeks before Sol Mamakwa went to Muskrat Dam to receive his vaccine, the community’s vaccine rate was 60%, but after his visit it spiked to 97% [25]. This suggests that it is critical for government and health care officials to engage with Indigenous community leaders when planning a mass public health intervention.

Many respondents reflected on the value of service-learning and how it is a path to social accountability. Most reflections focused on clinical courage, social accountability and cultural sensitivity, and how these learnings can be applied to future medical practice. Cultural sensitivity and social accountability can be taught especially well through service-learning. Service-learning has been shown to promote student engagement and learning, encourage health professionals to continue working in underserviced regions, and strengthen community relationships [16, 26]. Having medical learners return to these communities in their future medical practice and form trusting relationships with the community may promote social accountability and improve the quality of care the community receives [16]. Many of the medical learners reflected that the service-learning elective encouraged them to consider returning to these northern First Nation communities to locum in their future medical practice, but current research suggests that the longitudinal retention levels in northern communities are low [27]. This may be due to the remoteness of the communities, or the increased demand on physicians when working in low resource communities [27, 28]. One potential medical education solution is creating more longitudinal integrated clerkships in rural and remote communities for students who are from northern locations or of Indigenous descent. Research has shown that educating medical learners who are from the north in northern settings leads to a higher likelihood of them remaining and practicing in rural, remote, and northern communities [27].

The medical learners reflected on how the service-learning experience demonstrated the importance of providing high-quality, culturally competent care and more specifically how the CanMEDs competencies were key to making ORI successful. The CanMEDs competencies of Collaboration and Communication were especially relevant to this operation. Collaboration during this elective referred to working alongside other health professionals, patients, patients’ families, and the community members of the populations being served [29]. Communication consisted of working with Indigenous community members, ensuring culturally safe care for Indigenous patients, and dispelling any myths surrounding the COVID-19 vaccine. The medical learners were presented with a unique opportunity to learn and utilize the CanMEDs competencies first-hand. In-person service-learning has been shown to accelerate education and knowledge compared to in-classroom learning [30]. Because this service-learning occurred in-person within communities, partnership with community members and institutions was necessary. This fostered collaboration and communication between the medical learners and their surroundings and emphasized shared knowledge and learning to a greater degree [31]. Learning about the CanMEDs competencies and applying the competencies in a meaningful manner in real-time is important because it can enhance skills that will foster and promote the learners’ ability to deliver socially accountable healthcare in their future medical practice [26]. It is important to provide medical learners with service-learning experiences during their medical education for them to build a professional identity rooted in social accountability [31].

This paper may have been limited by the written reflections from which the data was collected, as the reflections were restricted to 500 words. However, due to the additional stress of COVID-19 on health professionals this length was deemed appropriate as it would not place an extra burden on participants. Another limitation of this study was that we were unable to collect descriptive or demographic (i.e., age, gender, ethnicity, year of study) data in accordance with the research ethics office. This omission may limit some of the interpretation of the data. NOSMU publishes basic demographic information about each Undergraduate class which may provide useful information about some of the participants in this study. Most of the Undergraduate student body at NOSMU consists of students from northern and/or rural Ontario [32]. The average age of the incoming Undergraduate medical learners at NOSMU is 25 years [32]. The final study limitation was that participation in this service-learning elective was self-initiated by the medical learners. These self-selected learners may be more keen to work and return to rural, remote or northern communities in their futures compared to their peers.

## Conclusion

Service-learning is an exceptional method which provides medical learners with an opportunity to expand their knowledge of the social determinants of health, social justice and interprofessional teamwork, while working with communities first-hand [26, 31]. It was evident in this study that learning medicine through a service-learning model led to a greater depth of knowledge on Indigenous health and culture and enhanced medical skills and knowledge. Future research should focus on longitudinal studies which follow the medical learners who participated in service-learning electives, to gain an understanding into whether service-learning promotes long-term retention of physicians in rural, remote, and northern communities. Future research on this topic could also be focused specifically on how service-learning can be implemented into the current medical educational landscape and the benefits of doing so.

## Data Availability

The datasets used and/or analysed during the study are available from the corresponding author upon reasonable request.

## Abbreviations

NOSMU: Northern Ontario School of Medicine University
ORI: Operation Remote Immunity
